# Association of body mass index, sagittal abdominal diameter and waist-hip ratio with cardiometabolic risk factors and adipocytokines in Arab children and adolescents

**DOI:** 10.1186/1471-2431-12-119

**Published:** 2012-08-07

**Authors:** Omar S Al-Attas, Nasser M Al-Daghri, Majed S Alokail, Khalid M Alkharfy, Hossam Draz, Sobhy Yakout, Shaun Sabico, George Chrousos

**Affiliations:** 1 Biomarkers Research Program, Biochemistry Department, College of Science, King Saud University, Riyadh, 11451, Kingdom of Saudi Arabia; 2 Center of Excellence in Biotechnology, King Saud University, Riyadh, 11451, Kingdom of Saudi Arabia; 3 Clinical Pharmacy Department, College of Pharmacy, King Saud University, Riyadh, 11451, Kingdom of Saudi Arabia; 4 Department of Biochemistry, National Research Centre, Cairo, 12311, Egypt; 5 First Department of Pediatrics, Athens University Medical School, Athens, 11527, Greece

**Keywords:** Sagittal abdominal diameter, Insulin resistance, Adipocytokines, Arab children

## Abstract

**Background:**

Sagittal abdominal diameter (SAD) is a novel anthropometric measure hypothesized to be a surrogate measure of visceral abdominal obesity in adults. This study aims to determine whether SAD is superior to other anthropometric measures such as body mass index (BMI) and waist to hip ratio (WHR) in terms of association to cardiometabolic risk and circulating adipocytokine concentrations in a cohort of Saudi children and adolescents.

**Methods:**

A total of 948 (495 boys and 453 girls) apparently healthy children with varying BMI, aged 10–17 years, were included in this cross sectional study. Fasting glucose, lipid profile, leptin, adiponectin, resistin, insulin, TNF-α and aPAI-1 were measured in serum and HOMA-IR was calculated. MetS components were defined according to the International Diabetes Federation (IDF) criteria.

**Results:**

BMI was superior to SAD as well as WHR, and had the highest number of significant associations to MetS components and adipocytokines even after adjustment for age and gender, including blood pressure, lipids, glucose and leptin.

**Conclusion:**

In conclusion, while SAD is significantly associated with components of MetS among children and adolescents, it is not superior to BMI. The use of SAD therefore may not be practical for use in the pediatric clinical setting. Follow-up studies are needed to determine whether SAD has clinical significance in terms of harder outcomes such as predicting diabetes mellitus or cardiovascular diseases.

## Background

Obesity confers increased risk for cardiovascular diseases as a result of accumulation of visceral fat, which alters intermediary metabolism and insulin sensitivity of peripheral tissues [1-5]. Conventionally, waist circumference is used in both clinical practice and biomedical research to measure abdominal obesity and to screen for the presence of the metabolic syndrome (MetS). Other anthropometric indices, such as body mass index (BMI), as well as the waist-hip ratio (WHR), are two of the most commonly used non-invasive biomarkers of obesity.

While BMI remains the most popular obesity measure, its main disadvantage and limitation over waist circumference and WHR is that it does not take into account body fat distribution, a major predisposing factor to metabolic abnormalities. However, a novel anthropometric index that can rival WHR and waist circumference in clinical risk assessment is the sagittal abdominal diameter (SAD), considered a reliable surrogate measure of visceral abdominal fat, the fat tissue type that is significantly associated with cardiometabolic risk variables such as obesity, dyslipidemia and hypertension [6-8], measured non-invasively by a simple caliper [9].

While several reports have suggested that SAD is a superior correlate of MetS criteria and insulin resistance than waist circumference, there has been little incorporation of this measure into routine clinical practice [10-12]. Furthermore, SAD was recently suggested to be superior to waist circumference in predicting dyslipidemia and hyperglycemia, at least in overweight adults [13]. The latter, measured at the level of the umbilicus, is modestly associated with coronary heart disease and visceral fat in males, at least in adults [4,14]. To date, very few studies have correlated SAD to other metabolic abnormalities, especially in children. Furthermore, very few studies have examined whether SAD in children and adolescents is superior to other anthropometric parameters in predicting cardiometabolic risk factors, particularly in this peninsula where increased prevalence of MetS and chronic diseases have been observed in both children and adults [15-17]. Thus, the present study aims to determine the superior marker of obesity through comparisons of SAD, BMI, WHR in terms of association strength with metabolic risk factors and levels of circulating adipocytokines in a cohort of Saudi children.

## Methods

A total of 948 (495 male and 453 female) Saudi children aged between 10 and 17 years, were randomly selected from the existing database of the Biomarkers Screening in Riyadh Program (RIYADH Cohort), a capital-wide study composed of randomly selected individuals from different Primary Health Care Centers (PHCCs) in Riyadh, KSA. Ethical approval was granted by the Ethics Committee of the College of Science Research Center, King Saud University (KSU), Riyadh, KSA. Written consent was obtained after study orientation. The study was carried out at the Biomarkers Research Program, KSU, Riyadh, KSA. A questionnaire focusing on demographic information and past medical history was given to all participating subjects. Children with acute co-morbidities that needed immediate medical attention were excluded from the study. MetS components were defined according to International Diabetes Federation (IDF) criteria [18].

### Anthropometry

Anthropometry included height (to the nearest 0.5 cm) and weight (to the nearest 0.1 kg), as well as waist and hip circumferences utilizing a standardized measuring tape in cm. The Holtain Khan abdominal caliper by Holtain Ltd (Crymych, UK) was used to measure SAD as previously described [19]. In brief, each subject was examined supine on a firm examination table. Using sliding calipers with parallel blades a direct reading can be made between its lower arm (touching the subject's back) and its sliding upper arm (touching the front of the subjects abdomen). BMI was calculated as kg/m2 and systolic and diastolic blood pressure measurements were obtained. The definition of BMI-based obesity employed is gender and age specific as proposed by Cole and colleagues [20], which provides cut off points for body mass index in childhood that was based on international survey and linked to the widely accepted adult cut-off points for overweight and obesity (BMI of 25 and 30 kg/m^2^, respectively).

### Blood chemistry

Participating subjects were requested to return to their respective PHCCs after an overnight fast for anthropometry and blood withdrawal. Blood was transferred immediately to a non-heparinized tube for centrifugation. Serum was then transferred to a pre-labeled plain tube, stored in ice, and delivered to the Biomarkers Research Program in KSU on the same day. Fasting serum samples were stored in a −20 °C freezer until analysis. Fasting glucose (FG) and lipid profile were measured using a chemical analyzer (Konelab, Vantaa, Finland). Serum leptin, adiponectin, resistin, insulin, TNF-α and aPAI-1 were measured using a Luminex instrument (Linco Research Inc., USA). The intra- and inter-assay variations were 1.4-7.9% and < 21%, respectively for the above mentioned parameters measured using Luminex multiplex assay. Minimum detectable concentrations (MDC) were: leptin, 85.4 pg/ml; adiponectin, 145.4 pg/ml; resistin, 6.7 pg/ml; insulin, 50.9 pg/ml; TNF-α, 0.14 pg/ml, and aPAI-1, 1.3 pg/ml. hsC-reactive protein was determined using enzyme-linked immunosorbent assays (ELISA) (Immunodiagnoztik AG, Germany) with an intra- and inter-assay variations of 5.5-6.0% and 11.6-13.8%, respectively. Serum angiotensin II was measured using human angiotensin II EIA kit (Phoenix pharmaceuticals, Belmont, CA, USA), (MDC 13 pg/ml; linear range 13–240 pg/ml) with intra- and inter-assay variations of 5.0-10.0% and < 15.0%, respectively. Homeostasis model assessment of insulin resistance (HOMA-IR) was calculated as fasting insulin (μU/mL) times fasting glucose (mmol/L)/22.5.

### Statistical analyses

Data were analyzed using the Statistical Package for the Social Sciences (SPSS for Windows, version 11.5). Data were expressed as mean plus/minus standard error. Group comparisons were done using one way Analysis of Co-variance (ANCOVA), with age and gender as covariates, and Bonferroni analysis *post-hoc*. Frequencies were expressed as percent (%). Partial (adjusted for age and gender) and bivariate (raw, unadjusted) correlation coefficients were determined and regression analysis was done to determine relations between variables of interest. Area under the curve (AUC) was done to reveal strength of associations elicited. *P*-values less than 0.05 were accepted to indicate statistically significant differences.

## Results

Table 1 shows the basic characteristics of subjects. The over-all prevalence of childhood obesity in this cohort was 11.7%. Figurem 1 shows the positive linear trend between SAD and age in both boys and girls as well as the mean SAD across ages. Mean SAD for girls reach a growth peak from ages 12–14, where the difference between boys is at its maximum, and starts to plateau at 15 years. Mean SAD for boys on the other hand starts to increase at 13 years of age and continues to rise modestly until 16 years (Figurem 1).

**Table 1:** General characteristics of the study population

**Parameters**	
N	948
Age (years)	13.7 ± 2.2
Obese (%)	11.7
Overweight (%)	16.7
Gender (Male/Female) (%)	52.2**/**47.8
Body Mass Index (kg/m^2^)	22.1 ± 6.3
BMI z-score	0.001*10^-13^ ± 1.0
Waist circumference (cm)	77.1 ± 20.5
Hip circumference (cm)	86.4 ± 19.8
Waist-Hip Ratio	0.91 ± 0.33
Sagittal Abdominal Diameter (cm)	17.4 ± 5.4
Sagittal Abdominal Diameter z-score	0.18 ± 0.67
Systolic Blood Pressure (mmHg)	106.4 ± 10.6
Diastolic Blood Pressure (mmHg)	69.5 ± 7.8
Glucose (mmol/l)	5.1 ± 1.3
Insulin (ng/ml)	11.2 ± 1.2
HOMA-IR	2.6 ± 0.28
Triglycerides (mmol/l)	1.1 ± 0.60
Total Cholesterol (mmol/l)	4.2 ± 0.85
LDL-Cholesterol (mmol/l)	2.7 ± 0.76
HDL-Cholesterol (mmol/l)	1.1 ± 0.35
Leptin (ng/ml)	15.7 ± 2.8
Adiponectin (μg/ml)	20.4 ± 1.4
Resistin (ng/ml)	18.6 ± 1.7
ANG II (ng/ml)	0.67 ± 0.05
aPAI-1 (ng/ml)	19.8 ± 2.4
C-Reactive Protein (μg/ml)	0.83 (2.8)
TNF-α (pg/ml)	8.6 ± 1.0

**Note:** Data represented by Mean ± Standard Error; CRP in median and inter-quartile range.

**Figure 1:**
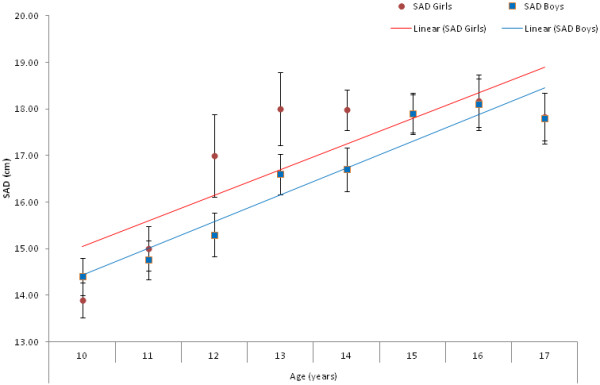
Linear trend between sagittal abdominal diameter in varying age groups in both boys and girls.

Bivariate and partial correlations between anthropometric indices and components of the metabolic syndrome, as well as inflammatory markers and adipocytokines are shown in Table 2. Bivariate associations of SAD showed significant associations to 12 out of 16 parameters examined; however, only blood pressure, triglycerides, HDL-cholesterol, leptin, resistin and aPAI-1 remained significant after adjusting for gender and age. BMI z-score on the other hand, had the highest number of significant associations, and retained blood pressure, lipids, glucose, and leptin after adjustment for age and gender. Finally, for WHR, only lipids (triglycerides, LDL- and total cholesterol), ANG II, as well as HOMA-IR remained significant after adjustment.

**Table 2:** Bivariate (Unadjusted) and partial (Controlled for Gender and Age) correlations of BMI, WHR and SAD to anthropometric and metabolic parameters

**PARAMETERS**	**BMI z-score**		**WHR**		**SAD z-score**	
	***Bivariate***	***Partial***	***Bivariate***	***Partial***	***Bivariate***	***Partial***
Systolic Blood Pressure (mmHg)	0.40 **	0.19 **	0.11	- 0.03	0.32 **	0.20 **
Diastolic Blood Pressure (mmHg)	0.29 **	0.18 **	- 0.01	- 0.03	0.25 **	0.16 **
Glucose (mmol/l)	0.19 **	0.18 **	0.07 *	0.06	0.09 **	0.02
Insulin (IU/ml)	0.52 **	- 0.01	0.17 *	0.10	0.38 **	- 0.10
HOMA-IR	0.52 **	- 0.01	0.21 **	0.12 *	0.36 **	- 0.10
Triglycerides (mmol/l)	0.30 **	0.21 **	0.11 *	0.23 **	0.17 **	0.08 *
Total Cholesterol (mmol/l)	0.13 **	0.14 **	- 0.02	- 0.11*	0.08 *	0.02
LDL-Cholesterol (mmol/l)	0.17 **	0.17 **	- 0.04	- 0.24 **	0.11	0.06
HDL-Cholesterol (mmol/l)	- 0.25 **	- 0.19 **	- 0.04	0.09	- 0.20 **	- 0.14 **
Leptin (ng/ml)	0.49 **	0.34 **	0.04	0.01	0.35 **	0.22 **
Adiponectin (μg/ml)	- 0.25 **	- 0.14	- 0.14	0.00	- 0.20 **	- 0.16
Resistin (ng/ml)	0.18 **	0.12	- 0.11 **	- 0.08	0.18 **	0.07 *
ANG II (ng/ml)	0.04	- 0.01	0.07	0.15 *	- 0.03	0.02
aPAI-1 (ng/ml)	0.13 **	0.14	0.02	- 0.00	0.10 **	0.10 **
C-Reactive Protein (μg/ml)	0.04	0.05	0.06	- 0.06	0.02	0.12
TNF-α (pg/ml)	−0.03	−0.50	−0.04	−0.42	−0.02	−0.31

**Note**: Data presented as coefficients (R); * denotes p-value < 0.05 level; ** denotes p-value < 0.01 level.

The area under the curve (AUCs) of various indices and metabolic risk components are summarized in Table 3. BMI z-score showed significance in all parameters measured with the exception of systolic blood pressure. SAD was a significant predictor of most other parameters, with the exception of systolic blood pressure and triglycerides. Lastly, waist-hip ratio was significant in glucose, 2 or more components of MetS and MetS itself.

**Table 3:** Areas under ROC curve (AUC) of various anthropometric indices and metS risk factors

**Test Variable**	**Anthropometric Parameter**	**AUC ± SE**	**Asymptotic Significance**	**95% Confidence Interval**
Systolic Blood Pressure	BMI z-score	0.526 ± 0.040	0.926	(0.447, 0.605)
	WHR	0.513 ± 0.001	0.094	(0.005, 0.031)
	SAD z-score	0.539 ± 0.048	0.893	(0.445, 0.633)
Triglycerides	BMI z-score	0.721 ± 0.065	**0.010**	(0.594, 0.848)
	WHR	0.662 ± 0.069	0.060	(0.527,0.798)
	SAD z-score	0.634 ± 0.080	0.119	(0.477, 0.792)
HDL-Cholesterol	BMI z-score	0.623 ± 0.019	**<0.001**	(0.583, 0.660)
	WHR	0.535 ± 0.020	0.070	(0.497, 0.574)
	SAD z-score	0.599 ± 0.019	**<0.001**	(0.561, 0.636)
Glucose	BMI z-score	0.615 ± 0.024	**<0.001**	(0.567, 0.662)
	WHR	0.563 ± 0.025	**0.012**	(0.515, 0.612)
	SAD z-score	0.554 ± 0.026	**0.031**	(0.504, 0.604)
2 or more IDF MetS Components	BMI z-score	0.777 ± 0.016	**<0.001**	(0.747, 0.808)
	WHR	0.641 ± 0.019	**<0.001**	(0.605, 0.678)
	SAD z-score	0.678 ± 0.019	**<0.001**	(0.641, 0.714)
IDF MetS	BMI z-score	0.776 ± 0.025	**<0.001**	(0.727, 0.825)
	WHR	0.632 ± 0.025	**<0.001**	(0.582, 0.681)
	SAD z-score	0.693 ± 0.029	**<0.001**	(0.636, 0.750)

**Note:** Significant at p < 0.05.

## Discussion

The main finding of the present cross-sectional study is that BMI was superior to both SAD and WHR in terms of significant associations to cardiometabolic parameters in children. SAD was no better than BMI, despite significant correlations with MetS components, insulin sensitivity indices and adipocytokine concentrations. Earlier studies done among children of European and Turkish descent showed that BMI was a better predictor of WHR and skin folds, respectively, with SAD values reported only as a correlate to BMI among European children [21,22].

Our findings however, differ from other ethnic groups, as waist circumference is more powerful in assessing metabolic disorders over other anthropometric values including SAD among Far Eastern children [23].

Our findings show that BMI is the best predictor of cardiometabolic risk factors as compared to SAD and WHR in children. With SAD however, some associations with cardiometabolic parameters remained significant even after adjustment for gender and age, notably blood pressure, triglycerides, and HDL-cholesterol. SAD also had more significant associations than BMI with regards to the adipocytokines suggesting that SAD may have a different predictive value independent of cardiometabolic risk factors. With the exception of HOMA-IR, these associations were not elicited with WHR. Furthermore, in terms of identifying adult patients with MetS, SAD, in the absence of imaging techniques, has a sensitivity of 91% and specificity of 80% in patients at risk for cardiovascular events [24], and even more superior in assessing cardiovascular risk among the severely obese [25]. It is noted however that the predictive utility of SAD among normal adults, and in this case, children, will be clearly less as compared to those who harbor known CVD risk factors such as obesity. In the present study, the significant influence of SAD on the expression of numerous adipocytokines makes it a better measure than WHR, but not as good as BMI, in assessing cardiometabolic risk profile in children. This is because when waist or hip circumference is measured in a standing person with an increased volume of intra-abdominal adipose tissue, all fat tissue is pulled towards gravity, and may therefore not be that accurate in assessing intra-abdominal fat especially among children who are very obese. When the same person lies supine the fat mass shifts cranially, causing anterior projection of the abdomen (abdominal height) which is measured by the SAD [19]. It is the antero-posterior fat that seems to be important for the prediction of the MetS. The persistence of significant associations of lipids and selected adipocytokines with SAD agrees with the findings of several cross-sectional observations that SAD is the surrogate marker for visceral fat as compared to waist circumference [26,27].

The authors acknowledge some limitations. The cross-sectional nature of the study limits our findings to observations and only prospective longitudinal studies can confirm whether the persistence of these associations translate into increased odds of developing chronic diseases. Pubertal status was also not assessed and this could have affected the results, since BMI, waist circumference, SAD and MetS itself can also be influenced by the developmental trajectories and hormonal changes occurring during adolescence, and excess adiposity during childhood could advance puberty in girls and delay onset in boys [28]. Finally, the results cannot be generalized, as it may differ if applied to children of other ethnicities.

## Conclusion

In conclusion, while SAD is significantly associated with several components of MetS, including adipcytokines in Arab children and adolescents, it is not superior to BMI in terms of association strength. The use of SAD, therefore, may not be clinically practical for routine use as compared to BMI, at least among children, but is more useful clinically when compared to waist-hip ratio. Prospective longitudinal studies are suggested to compare its causal association with harder endpoints such as early onset diabetes and cardiovascular disease.

## Competing interests

The authors declare that they have no competing interests.

## Authors’ contributions

OSA and NMA conceived and carried out the study. MSA and KA participated in the design, subject recruitments and data collection. HD and SY carried out sample analysis. SLS and GPC performed statistical analysis and drafted the final version of the manuscript. All authors approved and read the final manuscript.

## Pre-publication history

The pre-publication history for this paper can be accessed here:

http://www.biomedcentral.com/1471-2431/12/119/prepub
